# Improving the *Caenorhabditis elegans* Genome Annotation Using Machine Learning

**DOI:** 10.1371/journal.pcbi.0030020

**Published:** 2007-02-23

**Authors:** Gunnar Rätsch, Sören Sonnenburg, Jagan Srinivasan, Hanh Witte, Klaus-R Müller, Ralf-J Sommer, Bernhard Schölkopf

**Affiliations:** 1 Friedrich Miescher Laboratory, Max Planck Society, Tübingen, Germany; 2 Fraunhofer FIRST, Berlin, Germany; 3 Division of Biology, California Institute of Technology, Pasadena, California, United States of America; 4 Max Planck Institute for Developmental Biology, Tübingen, Germany; 5 Computer Science Department, Technical University of Berlin, Berlin, Germany; 6 Max Planck Institute for Biological Cybernetics, Tübingen, Germany; Duke University, United States of America

## Abstract

For modern biology, precise genome annotations are of prime importance, as they allow the accurate definition of genic regions. We employ state-of-the-art machine learning methods to assay and improve the accuracy of the genome annotation of the nematode Caenorhabditis elegans. The proposed machine learning system is trained to recognize exons and introns on the unspliced mRNA, utilizing recent advances in support vector machines and label sequence learning. In 87% (coding and untranslated regions) and 95% (coding regions only) of all genes tested in several out-of-sample evaluations, our method correctly identified all exons and introns. Notably, only 37% and 50%, respectively, of the presently unconfirmed genes in the C. elegans genome annotation agree with our predictions, thus we hypothesize that a sizable fraction of those genes are not correctly annotated. A retrospective evaluation of the Wormbase WS120 annotation [1] of C. elegans reveals that splice form predictions on unconfirmed genes in WS120 are inaccurate in about 18% of the considered cases, while our predictions deviate from the truth only in 10%–13%. We experimentally analyzed 20 controversial genes on which our system and the annotation disagree, confirming the superiority of our predictions. While our method correctly predicted 75% of those cases, the standard annotation was never completely correct. The accuracy of our system is further corroborated by a comparison with two other recently proposed systems that can be used for splice form prediction: SNAP and ExonHunter. We conclude that the genome annotation of C. elegans and other organisms can be greatly enhanced using modern machine learning technology.

## Author Summary

Eukaryotic genes contain introns, which are intervening sequences that are excised from a gene transcript with the concomitant ligation of flanking segments called exons. The process of removing introns is called *splicing*. It involves biochemical mechanisms that to date are too complex to be modeled comprehensively and accurately. However, abundant sequencing results can serve as a blueprint database exemplifying what this process accomplishes. Using this database, we employ discriminative machine learning techniques to predict the mature mRNA given the unspliced pre-mRNA. Our method utilizes support vector machines and recent advances in label sequence learning, originally developed for natural language processing. The system, called *mSplicer,* was trained and evaluated on the genome of the nematode *C. elegans,* a well-studied model organism. We were able to show that *mSplicer* correctly predicts the splice form in most cases. Surprisingly, our predictions on currently unconfirmed genes deviate considerably from the public genome annotation. It is hypothesized that a sizable fraction of those genes are not correctly annotated. A retrospective evaluation and additional sequencing results show the superiority of *mSplicer'*s predictions. It is concluded that the annotation of nematode and other genomes can be greatly enhanced using modern machine learning.

## Introduction

C. elegans is a free-living soil nematode with a cosmopolitan distribution. Its short life cycle, self-fertilizing propagation, simple anatomy, and the ease of genetic and experimental manipulations made C. elegans an important model system in biology. Today, C. elegans is one of the best-studied organisms in experimental biology. Its genome is about 100 million base pairs in size, organized in five autosomes and one sex chromosome and was the first metazoan genome to be sequenced from end to end [2]. A recent release of the C. elegans genome (WS150, [3]) has an estimated 22,858 genes when including the alternatively spliced forms. Only 6,513 (28.5%) genes have been fully confirmed by cDNA and EST sequences, i.e., by sequenced parts of mRNA. Of the remaining 16,345 gene models, primarily based on computational predictions, 11,417 (49.9%) have been partially confirmed and 4,928 (21.6%) lack transcriptional evidence.

Eukaryotic genes contain introns, which are intervening sequences that are excised from a gene transcript with the concomitant ligation of flanking segments called exons. The process of removing introns is called *splicing,* which involves biochemical mechanisms that to date are too complex to be modeled comprehensively and accurately. However, abundant sequencing results can serve as a blueprint database exemplifying what this process accomplishes.

In the present work, we employ machine learning techniques to model and predict how the splicing process acts. (We only consider splice forms that are nonalternative and canonical or standard noncanonical, i.e., exhibit the GT or GC at the donor site and AG consensus at the acceptor site.) Our goal is to learn to simulate the biological process generating mature mRNA from unspliced pre-mRNA, given a sufficient number of examples for “training.” For detecting the donor and acceptor splice sites, as well as for recognizing the exon and intron content, we employ support vector machine (SVM) classifiers [4–6], which have been used with considerable success in a variety of fields including computational biology [7–10].

**Figure 1:**
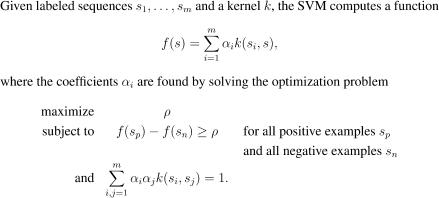
Simplified Support Vector Machine Learn a function *f* such that the difference of predictions (the *margin*) of positively and negatively labeled examples is maximal. Previously unseen examples will often be close to the training examples. The large margin then ensures that these examples are correctly classified as well, i.e., the decision rule *generalizes*.

**Figure 2:**
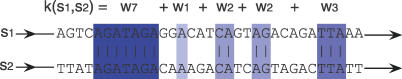
Given Two Sequences, *s*_1_ and *s*_2_ of Equal Length, Our Kernel Consists of a Weighted Sum to Which Each Match in the Sequences Makes a Contribution *w*_l_ Depending on Its Length *l,* Where Longer Matches Contribute More Significantly For predictions, we use a window of 140 nt around the potential splice site (cf. Materials and Methods for details, including the procedure of how the length of the window is determined).

**Figure 3:**
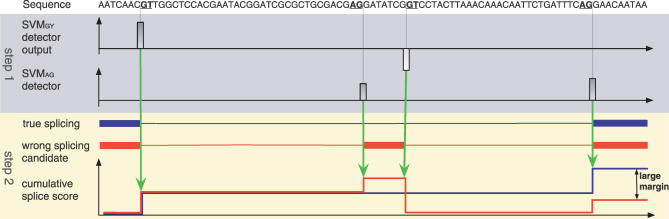
Given the Start of the First and the End of the Last Exon, Our System (*mSplicer*) First Scans the Sequence Using SVM Detectors Trained To Recognize Donor (SVM*_GY_*) and Acceptor (SVM*_AG_*) Splice Sites The detectors assign a score to each candidate site, shown below the sequence. In combination with additional information including outputs of SVMs recognizing exon/intron content, and scores for exon/intron lengths (unpublished data), these splice site scores contribute to the cumulative score for a putative splicing isoform. The bottom graph (step 2) illustrates the computation of the cumulative scores for two splicing isoforms, where the score at end of the sequence is the final score of the isoform. The contributions of the individual detector outputs, lengths of segments, as well as properties of the segments to the score are adjusted during training. They are optimized such that the *margin* between the true splicing isoform (shown in blue) and all other (wrong) isoforms (one of them is shown in red) is maximized. Prediction of new sequences works by selecting the splicing isoform with the maximum cumulative score. This can be implemented using dynamic programming related to decoding generalized HMMs 12, which also allows one to enforce certain constraints on the isoform (e.g., an open reading frame).

**Figure 4:**
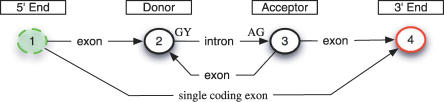
An Elementary State Model for Unspliced mRNA The 5′ end of the transcript is either directly followed by the 3′ end (single exon gene) or by an arbitrary number of donor–acceptor splice site pairs exhibiting the GT/GC and AG dimmer. A transition in this state model corresponds to *accepting* a whole segment (as in generalized HMMs 12), i.e., an exon or intron, with the corresponding dimer at the 3′ boundary of the segment (except in state 4).

**Figure 5:**
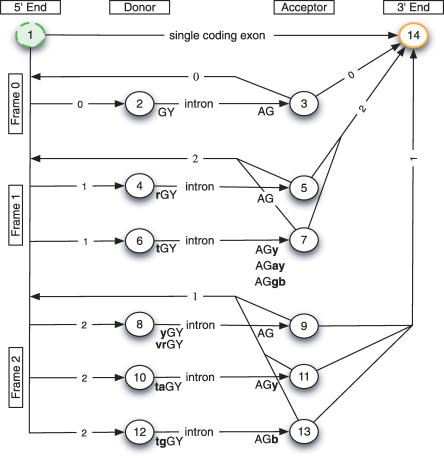
The State Model That Uses Open Reading Frame Information The sequences next to the state indicate which consensus has to appear at the transitions between intron (capital) and exon (bold). Here, we use the IUPAC code for ambiguous nucleotides (e.g., B = C/G/T, R = A/G, Y = C/T). The digit on the transition arrows is related to the reading frame and indicates the required frame shift to follow the transition (e.g., between state 1 and 2, one can only accept exons leading to a frame shift of 0). Also, it defines in which frame stop codons are allowed to occur—no stop codon should appear in-frame. Finally, the model is constructed such that in-frame stop codons cannot be assembled on the exon boundaries (this required the three additional state pairs 6/7, 10/11, and 12/13).

SVMs have their mathematical foundations in a statistical theory of learning and attempt to *discriminate* two classes by separating them with a large margin (“margin maximization”). SVMs are trained by solving an optimization problem (Figure 1) involving labeled training examples—true splice sites (positive) and decoys (negative). They employ similarity measures referred to as *kernels* that are designed for the classification task at hand. In our case, the kernels compare pairs of sequences in terms of their matching substring motifs [9,11,12] as illustrated in Figure 2 (cf. Material and Methods for more details). The idea of our algorithm is to first scan the unspliced mRNA using the SVM-based splice site detectors. In a second step, their predictions are combined to form the overall splicing prediction (cf. Figure 3 as well as Materials and Methods for details). This is implemented using a state-based system similar to standard hidden Markov model (HMM)–based gene-finding approaches [13–18]. We consider two different models: the simpler model implements the general rule that the start of the sequence is followed by a number (≥0) of donor and acceptor splice site pairs (5′ and 3′ ends of the intron) before the sequence ends (cf. Figure 4). If, moreover, one assumes the start and end of the *coding region* to be given, one can exploit that the spliced sequence consists of a string of non-stop *codons* terminated by a stop *codon* (TAA, TAG, TGA). In this case, the sum of the lengths of the coding parts of exons is divisible by three and the sequence does not contain in-frame stop codons. This can be translated into an alternative, more sophisticated model (cf. Figure 5) that is expected to perform better on coding regions, and may provide false predictions otherwise. The simpler model, on the other hand, is also applicable to untranslated regions (UTR); if in doubt, one should thus resort to this model.

The main difference of our approach from HMM-based gene-finding approaches (e.g., [14]) is that the parameters are obtained by using a *discriminative* machine learning method originally developed in the fields of natural language processing and information retrieval [19]. Instead of estimating probabilities with HMMs, we estimate a function that ranks splice forms such that the true splice form is ranked highest—with a large margin to all other splice forms. As all steps in our system are heavily based on the above-mentioned concept of margin maximization, we refer to it as *margin splicer* (*mSplicer*).

## Results

### Prediction Accuracy on Unseen Sequences

For our evaluation, we distinguish two cases: (a) the most general and difficult case *“UCI”* where the pre-mRNA sequence may include UTRs, coding regions, as well as introns; and (b) the case where we assume the start and stop codons are given and the sequence only consists of coding regions and introns *(“CI”)*. In the *UCI* setting, we used the EST-extended WS120 cDNA sequences (see above) for testing (1,177 sequences, including 27 with GC donor splice sites). Only the subsequences between the annotated start and end of coding regions (if known and valid) were included in the *CI* set (1,138 sequences, including 27 with GC donor splice sites). In both sets we excluded loci showing evidence for alternative splicing and unusual noncanonical splice sites.

On the *UCI* set, we used our method based on the simple model outlined as in Figure 4, referred to as SM. It predicted all splice sites correctly in 1,023 out of 1,177 cases (13.1% error rate). For the *CI* set, we used the more sophisticated model taking advantage of ORF information outlined in Figure 5, referred to as OM. Here, 1,083 out of 1,138 cases were predicted correctly (4.8% error rate). A summary of these results are given in Table 1.

**Table 1:**
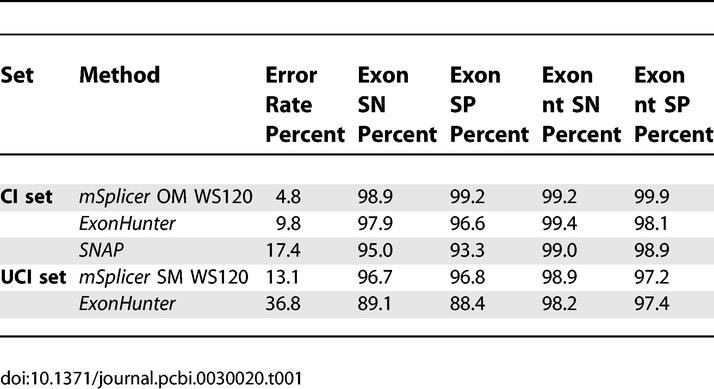
Splice Form Error Rates (1-Accuracy), Exon Sensitivities, Exon Specificities, Exon Nucleotide Sensitivities, Exon Nucleotide Specificities of *mSplicer*—with (OM) and without (SM)—Using ORF Information as well as *ExonHunter* and *SNAP* on Two Different Problems: mRNA Including (UCI) and Excluding (CI) UTR

For comparison, we tested two recently proposed state-of-the-art gene-finding systems, *SNAP* [20] and *ExonHunter* [21], adapted to the problem of splice form prediction. (*SNAP* was trained by its author on a set that was overlapping with our test sets; hence, the estimated error rates are expected to be lower than they would be when trained on our training set. *ExonHunter* is a comprehensive gene finder that can use many experimental sources of information. Here we only tested its HMM-based *ab initio* core trained by its authors on the same training set as *mSplicer*.) For evaluation we excluded cases with noncanonical splice sites since *SNAP* and *ExonHunter* cannot predict them. They achieve error rates of 17.4% and 9.8% on the *CI* set using ORF information. For *ExonHunter,* we were able to obtain predictions of a modified version (by the authors of *ExonHunter*) that does not take ORF information into account. (The system used was trained, however, on coding regions and using it on UTRs may significantly affect its performance.) In that case, the error rate on the *UCI* set is considerably higher: 36.8%. These results show that *mSplicer* greatly outperforms both methods, which is even more remarkable as *mSplicer* solves the more difficult task of including GC introns in the predictions: 23 *(UCI)* or 25 *(CI)* out of 27 cases with a GC splice site were predicted correctly, respectively. For simplicity of the following presentation, we exclude cases with GC splice sites in all of the subsequent analyses. For completeness, in Table 2 we also provide an evaluation of *mSplicer* trained and evaluated on sequences derived from WS150.

**Table 2:**
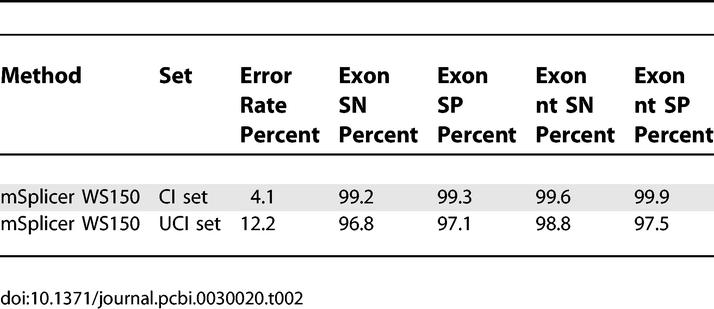
Splice Form Error Rates, Sensitivities, and Specificities of *mSplicer* Trained on WS150 (Including Signal and Content Sensors)

### Retrospective Evaluation of the Wormbase Annotation

Comparing the splice form predictions of our methods with the WS120 annotation on completely unconfirmed genes, we find disagreements in 62.5% (SM) or 50.0% (OM) of such genes, respectively. The results are summarized in Table 3. (As before, we excluded alternatively spliced genes and those that have noncanonical splice sites. Moreover, we used the annotated start and end of the coding region.) Based on these numbers and assuming that on this set our methods perform as well as reported above, one could conclude that the WS120 annotation is rather inaccurate on yet *unconfirmed genes*. (Note that if *mSplicer* with ORF information got 5% of the cases wrong, while disagreeing in 50% of the cases with the annotation, then the annotation would be wrong or at least incomplete in at least 45% of the cases.) Such a conclusion would be well in line with an independent whole genome analysis that showed that at least 50% of the predicted unconfirmed genes needed correction in their intron/exon structure [22]. However, the frequent disagreement can also be partially explained by inclusion of alternatively spliced genes. In the latter case, it is conceivable that both systems predict a valid splice form yet still disagree.

**Table 3:**
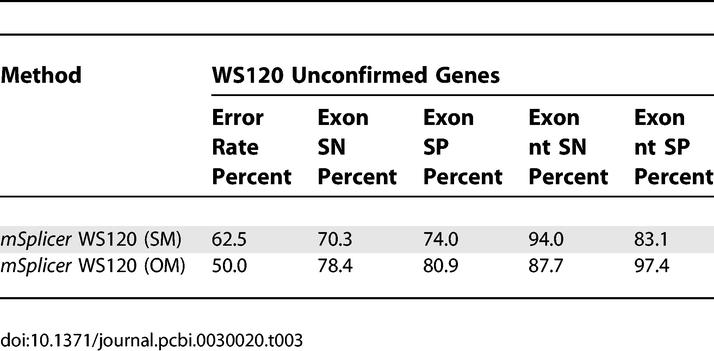
Measure of the Agreement of the WS120 Annotation on 5,166 Completely Unconfirmed Genes with *mSplicer'*s Predictions (SM and OM) (Reusing WS120′s Gene Starts and Ends)

One way of using our algorithm is to let it predict the splice form using the annotated 5′ and 3′ ends. Given our results, we expect that the resulting new annotation is considerably more accurate. To objectively evaluate this approach, we compare the accuracy of the WS120 annotation and our predictions based on WS120. For the evaluation, we use new cDNA and EST sequences that have been published in the databases between the publication dates of WS120 and WS150 as an independent test set: after aligning them to the genomic sequence [23] and identifying novel confirmed exons and introns, we determine overlapping segments between previously unconfirmed genes and the newly EST confirmed exons and introns. (Note that these segments are on average much shorter than complete genes and may include alternatively spliced exons and introns.) The new splicing information agrees with the WS120 annotation only in 259 out of 428 of these segments (error rate 39.5%). Often the WS120 annotation was wrong at the 5′ or 3′ end of the gene (merged or split genes). We therefore consider shortened segments such that there is an agreement at the terminal ends between the annotation, our predictions, and the new EST information. We find that the WS120 annotation agrees on 348 of the 424 segments (error rate 17.9%), while *mSplicer* agrees in 370 (SM) and 380 (OM) cases (error rates 12.7% and 10.4%, respectively). The results are summarized in Table 4. When interpreting these results, it should be borne in mind that the annotation is usually improved manually, which is known to improve the quality of genome annotations, whereas our result is obtained fully automatically.

**Table 4:**
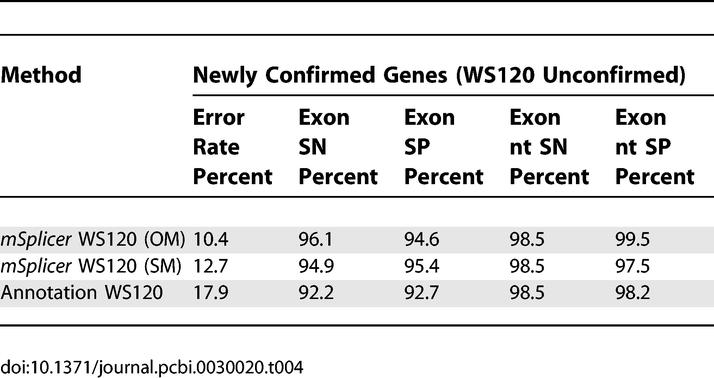
On Newly Confirmed Segments, Measure of the Accuracy of the WS120 Annotation and *mSplicer* Based on WS120 (OM and SM)

### Application to the C. elegans Genome Annotation

We can now use *mSplicer* to improve the current annotation of C. elegans. We generated predictions based on Wormbase annotation WS160, where we let *mSplicer* predict within the boundaries of annotated transcripts. As before, we separately analyzed the mixed regions (from annotated transcription start to end using model SM) and the coding regions (from annotated translation start to end using model OM). The new annotation is available for download in GFF format at http://www.fml.mpg.de/raetsch/projects/msplicer. Additionally, it is available on the Wormbase development website http://www.wormbase.org (tracks mSplicer and mSplicer-ORF).

We have compared *mSplicer*'s predictions with the WS160 annotation on genes that have not been used for training of our method. Depending on the confirmation status of a gene, we get varying levels of agreement with the WS160 annotation, which are reported in Table 5. The large agreement on confirmed genes corroborates the high performance of our method (this includes genes with alternative transcription starts and ends). The strong disagreement for alternatively spliced genes stems from the fact that mSplicer cannot predict alternative isoforms. However, the significant disagreement between the WS160 annotation and our predictions on partially confirmed as well as unconfirmed genes is likely to be due to inaccuracies in the current annotation.

**Table 5:**
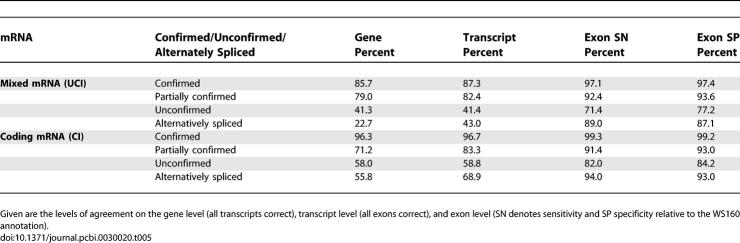
Comparison between Wormbase Annotation WS160 and the One Generated by *mSplicer* Applied to Annotated Transcripts

### Verification of Unconfirmed Genes by RT–PCR

We performed biological experiments on 20 unconfirmed genes randomly chosen from those where the *mSplicer* predictions differed significantly from the WS120 annotation. Primers were designed to amplify and sequence the parts of mRNAs of interest (cf. Materials and Methods as well as Protocol S1 for details). By aligning the sequenced cDNA to genome [23], we identified the true splice sites. The predictions of *mSplicer* without ORF information were completely correct in 15 out of the 20 cases (error rate 25%), while the WS120 annotation never exactly matched all new splice sites. Note that this figure (25%) is higher than our system's estimated error rate (13.1%), which we largely attribute to the fact that a biased (“hard”) set of particularly difficult genes has been chosen (the ones on which our system significantly disagrees with the annotation).

Protocol S1 contains illustrations comparing the sequencing results with the annotation and our predictions. We observed that if our predictions deviated from the sequencing results, then it was a complete exon or intron that was missing or superfluous. This indicates that the splice form predictors work very well, but there might be additional and undetected regulatory effects leading to the inclusion or exclusion of the exons or introns. For the WS120 annotation, we found many additional ways of how it deviated from the sequencing results, including mistakes at only one of the two splice sites.

### Analysis of the Splice Site Recognizers

**Figure 6:**
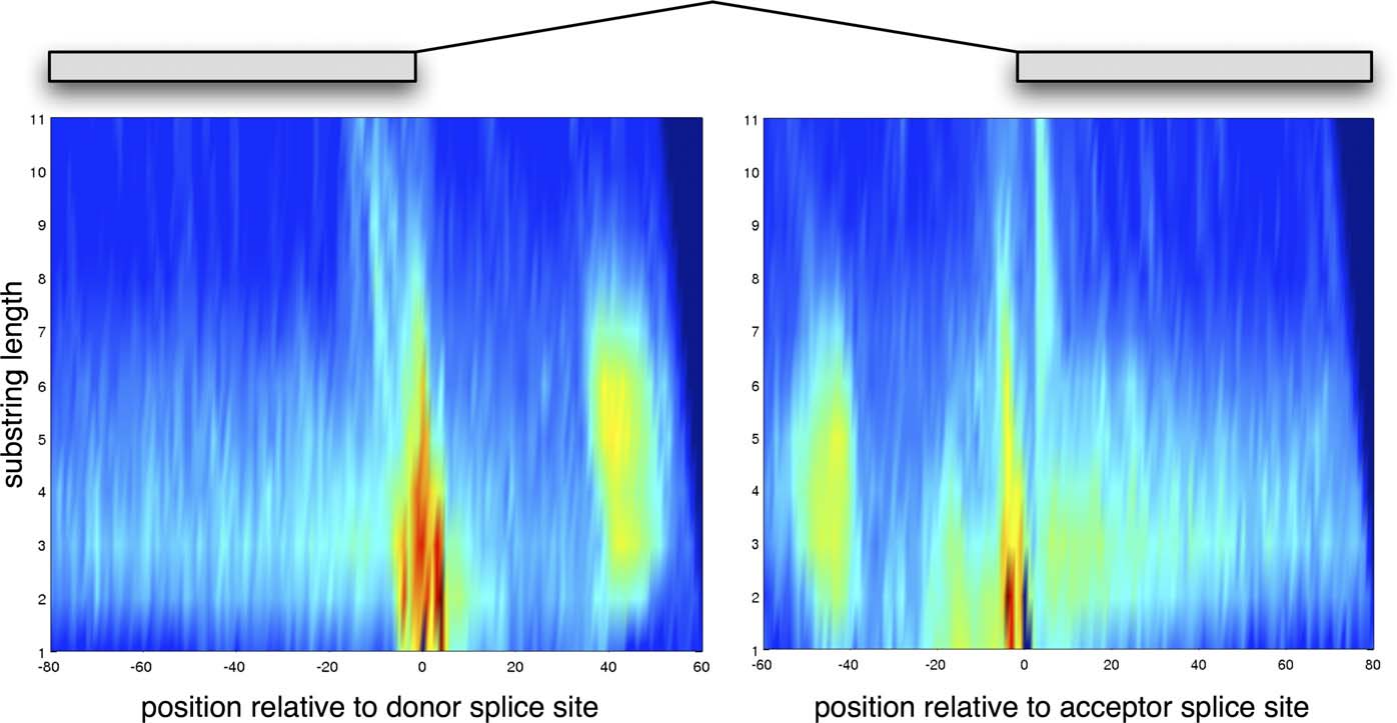
POIMs for Donor (Left) and Acceptor (Right) SVM Classifiers Shown are the color-coded importance scores of substring lengths for positions around the splice sites. Near the splice site, many important oligomers are identified. Particularly long substrings are important upstream of the donor and downstream of the acceptor site. See the main text for discussion.

One important difference of our method compared with previous approaches is the use of a similarity measure between sequences that takes the co-occurrence of long substrings into account. For the splice site, signal detectors strings up to length 22 and for the content sensors strings of up to length six, were considered. Techniques such as *SNAP* or *Genscan* [13] typically rely on much shorter substrings while using position-specific scoring matrices (PSSMs) for splice sites and second-order Markov models for exon/intron content. We found that for splice site detection in *C. elegans,* position-specific scoring matrices are not sufficient. If we allow long substrings to contribute, we can significantly improve the recognition performance. To illustrate this, we measured the area under the precision recall curve (auPRC, cf. [24]) for the SVM splice site classifiers while restricting the maximal length *d* of considered substrings. We found that the auPRC for classifying acceptor (donor) site with *d* = 1 is only 79.9% (62.2%). The performance increases when increasing *d*—for instance for *d* = 2 to 93.0% (89.7%)—and reaches a plateau for *d* = 8 at 95.9% (93.9%). To gain insights into what the SVM uses for discrimination, we study so-called *positional oligomer importance matrices* (POIMs) [25,26] that illustrate which length of substrings is important at which position (cf. Materials and Methods for details). Figure 6 shows the POIMs for donor (left) and acceptor (right) splice sites. We can observe that there are two regions per site that are of importance: near the splice site and around 50 nucleotides (nt) downstream or upstream. It turns out that introns are often rather short (only 50nt) and the weaker site relates to sequence signals of the other splice site. We find that the intronic regions near the splice sites are of particular importance, which is in line with the current understanding of how splicing works. Finally, we find that near the end (10–20 nt upstream of donor site) and at the start (2–6 nt downstream of acceptor site) of the exon very long substrings are important for discrimination, which are likely to correspond to exonic splicing enhancer or inhibitor binding sites (see [27] and references therein). A list of the most important substrings is listed in Protocol S1.

### Predictions on Other Nematode Genomes

We studied how well *mSplicer* trained on C. elegans generalizes to other nematode genomes. We collected all available EST sequences for Caenorhabditis briggsae [28], Caenorhabditis remanei [29], and Pristionchus pacificus [30], and used them as before for an out-of-sample evaluation (see Protocol S1 for details of the data preparation; for P. pacificus we only used 750 of the 2,952 splice forms for evaluation). The results of the evaluation are summarized in Table 6. We observe that the exon sensitivity and specificity for C. briggsae and C. remanei (95.1% to 96.3%) is only slightly lower than for C. elegans (96.7% and 96.8%). The performance of *mSplicer* is drastically lower for P. pacificus. One reason is the significantly different intron and exon length distribution that we observe in P. pacificus. We therefore trained two additional versions: (a) we use level 1 as trained on C. elegans and only retrain level 2 using 500 EST-confirmed splice forms (“*mSplicer* WS120/P. pac.”) and (b) fully retrain both levels using 1,702 and 500 EST-confirmed splice forms (“*mSplicer* P. pac.”), respectively. We find that retraining level 2 alone almost reaches the exon prediction accuracy of C. elegans. Additionally retraining level 1 does not lead to much further improvement. For C. briggsae and *C. remanei,* retraining did not lead to significant improvements (unpublished data).

**Table 6:**
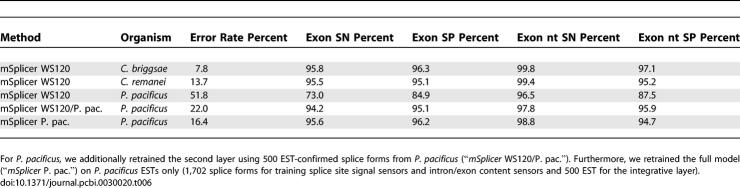
Error Rates, Sensitivities, and Specificities of *mSplicer* (SM) for Three Other Nematodes Trained on C. elegans Sequences (“*mSplicer* WS120”)

Finally, we repeated the retrospective analysis for the C. briggsae genome annotation. We identified 489 newly EST-confirmed segments that matched the *cb25* annotation. We evaluated how well the annotation and both versions of *mSplicer* performed on these segments. The results are summarized in Table 7. It should be noted that the gene error rates are smaller than before, since the segments are much shorter than whole genes.

**Table 7:**
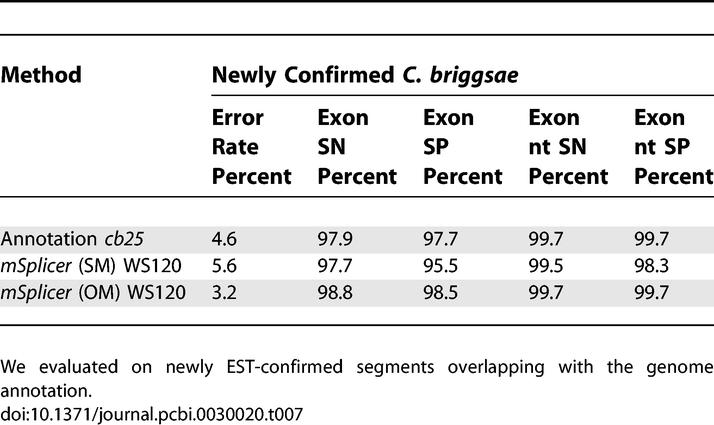
Error Rate, Sensitivities, and Specificities of the *cb25* Genome Annotation of the C. briggsae Genome and *mSplicer* Trained on WS120

### Summary of Results

Concluding from the presented three comparisons for *C. elegans,* we note that *mSplicer* significantly improves both over the existing annotation and over state-of-the art splice form predictors such as *SNAP* or *ExonHunter*. Each of the comparisons contribute a different piece of information to this conclusion: (a) 5%–13% error rates achieved on a very clean set of cDNA confirmed genes, (b) 10%–13% error rates in the retrospective analysis, and finally (c) a 25% error rate in the reverse transcription polymerase chain reaction (RT–PCR) validation experiments on a biased hard set of genes. In all cases, *mSplicer'*s error rates were at least 40% smaller than those of the other methods we compared with.

## Discussion

Our results show that on unconfirmed genes, our method can significantly improve the annotation. This is all the more remarkable since we only use information which in principle is also available to the cellular splicing machinery, such as sequence-based splice site identification (e.g., available via the splicing factors U1–U6), lengths of exons and introns (via physical properties of mRNA), and intron as well as exon content (for instance, via splice enhancers). We do not use exon counts, repeat masking, similarity to known genes and proteins, or any other evolutionary information. This distinguishes our method from alignment-based systems which do not put an emphasis on statistical structure and learning, but typically rely entirely on homology and evolutionary information [31–36]. The fact that *mSplicer* mainly relies on very accurate splice site predictions explains why *mSplicer*'s prediction accuracy is very high and also why it does not decay drastically in UTRs (unpublished data). It is to be noted that additional information, however, could complement our predictions. Closer in spirit to our machine learning approach are systems such as *Genscan* [14], *SNAP* [20], or *ExonHunter* [21] that are used in many genome annotations. However, these systems are typically based on generative models, trying to estimate probability densities. It has been argued that approaches of this type are not necessarily tuned to produce the best *discrimination,* as high-dimensional density estimation is known to be a task harder than discrimination, thus density estimation can be seen as a detour forcing generative approaches to solve a problem harder than necessary [4]. We conjecture that the key to success of our method lies in the fact that all parts of the *mSplicer* system were trained using discriminative learning techniques.

While interpreting our results, it should be noted that since C. elegans is one of the best-studied model systems, its annotation is expected to be more accurate than those of less well-studied or more complex organisms. Systems such as ours thus also offer hope towards a better annotation for these genomes [22]. In addition, our approach can be applied to genomes where only a small fraction of sequenced mRNA is available. For instance, for P. pacificus there are only relatively few EST sequences available. Statistical properties of the P. pacificus genome deviated considerably from those of C. elegans genome (e.g., exons and introns are on average only half as long). Hence, it is not surprising that the error rates of *mSplicer* are considerably higher than for C. elegans. However, after partly retraining our C. elegans system, *mSplicer* (SM) achieved an error rate of only 22%. For the much closer relatives C. briggsae and C. remanei, *mSplicer* based on WS120 already turned out to be very accurate in predicting splice forms. These observations illustrate both the universality of the splicing mechanism in nematodes and the strengths of our approach.

## Materials and Methods

### Preparation of sequence data and evaluation.

Following a statistical setup common in machine learning, we trained our system on 60% of the available cDNA sequences currently known for C. elegans (based on Wormbase 3, version WS120). The remaining 40% of the cDNA sequences were used to generate an independent set for out-of-sample testing. Additionally, we used available EST sequences (dbEST [37], as of 19 February 2004) to maximally extend the cDNA sequences at the 5′ and 3′ ends. For training and validation we did not use any EST sequences overlapping with the 40% of the cDNA sequences for out-of-sample prediction.

The methodology of learning a model on a training set, tuning the model parameters on a validation set, and finally using this fixed model on the test set for an out-of-sample prediction, is common in statistics and machine learning. The out-of-sample prediction yields an unbiased estimate for the overall prediction quality of the system, provided that the underlying statistical distribution of the test set is representative for the data-generating process.

### Identification of splice sites.

From the set of EST sequences not overlapping the validation and test set, we extracted sequences of confirmed donor (intron start) and acceptor (intron end) splice sites. For acceptor splice sites, we used a window of 80 nt upstream to 60 nt downstream of the site. For donor sites, we used 60 nt upstream and 80 nt downstream. Also from these training sequences we extracted non-splice sites, which are within an exon or intron of the sequence and have AG (acceptor) or GT/GC (donor) consensus. We train a SVM [4] with soft-margin using the so-called “weighted degree” kernel [10, 24]. The kernel mainly takes into account positional information (relative to the splice site) about the appearance of certain motifs (distinguishing it from the spectrum kernel used for the content sensors). It computes the scalar product between two sequences s and s′:

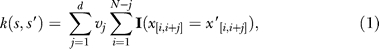
where *N* = 140 is the length of the sequence and *x*_[*a*,*b*]_ denotes the substring of *x* from position *a* to (excluding) *b*. Moreover, **I**(*true*) = 1, **I**(*false*) = 0, and *v_j_*: = *d* − *j* + 1. We used a normalization of the kernel 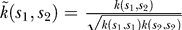 and *d* = 22 for the recognition of splice sites. Additionally, the regularization parameter of the SVM was set to be *C* = 2 and *C* = 3 for acceptor and donor sites, respectively. All parameters (including the window size) have been tuned on the validation set. For SVM training, we used the freely available software package *SHOGUN* developed by some of the authors [25,38] (available for download from http://www.shogun-toolbox.org). SVM training resulted in 61,233 and 79,000 support vectors for detecting acceptor and donor sites, respectively. The ROC scores (area under the receiver operator curve) for the resulting classifiers on the test set are 99.62% (acceptor) and 99.74% (donor). The auPRC are 96.29% (acceptor) and 94.38% (donor).

To generate the POIMs, we compute the contributions of *k*-mers with 1 ≤ *k* ≤ *d* to all *d~*-mers starting at position *p* = 1,...,*N,* where we used *d* = 22 and *d~* =1,...,11. The idea is to identify all *k*-mers with 1 ≤ *k* ≤ *d* overlapping with the *d~*-mers of the trained SVM classifier. The weights of the overlapping *k*-mers are then marginalized, summed up, and assigned to the identified *d~*-mers. This leads to a weighting for *d~*-mers **u** for each position in the sequence: W**_u_**_,p_, which may be summarized by *S_*d~*,p_* = max**_u_**(*W* **_u_**_,*p*_). We compute this quantity for *d~* = 1,…,11 leading to the two 11 × 141 matrices displayed in Figure 6. Note that the above computation can be done efficiently using index data structures implemented in *SHOGUN* and described in detail in [26].

### Identification of exon and intron content.

To obtain the exon content sensor, we derived a set of exons from the ESTs not overlapping the validation or test set. As negative examples, we used subsequences of intronic sequences sampled so that both sets of strings have roughly the same length distribution. We trained an SVM using the Spectrum kernel [12] of degree *d* = 3 to *d* = 6, where we count occurring *d*-mers only once and used *C* = 1 as regularization parameter. The model parameters have been obtained by tuning them on the validation set. We used a normalization of the kernel 
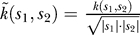
 , where |*s*| is the length of the sequence. We proceeded analogously for the intron content sensor.

### Integration.

The idea is to learn a function that assigns a score to a splice form such that the true splice form is ranked highest while all other splice forms have a significantly lower score. The function depends on parameters that are determined during training of the algorithm. In our case it is defined in terms of several functions determining the contributions of the content sensors (*f_E_*_,*d*_ and *f_I_*_,*d*_), the splice site predictors (*S_AG_* and *S_GY_*), and the lengths of introns and exons (*S_L_I__ , S_L_E__ ,  S_L_E,s__ ,  S_L_E, f__* , and *S_L_E,l__*).

We assume that the start of the first exon *p_s_* and the end of last exon *p_e_* are given. Then a splice form for a sequence *s* is given by a sequence of donor–acceptor pairs 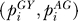 . The *cumulative splice score* 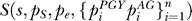 for a sequence *s* was computed as follows:

— If there is only a single exon, i.e., *n* = 0, then

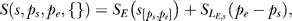
where *s*_[a,b]_ is the subsequence of *s* between positions *a* and *b,* 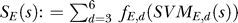 is the score for the exon content, and *S_L_E_,s_*(*l*) is the score for the length *l* of a single exon, whereby *SVM_E_*_,*d*_(*s*) is the output of the exon content sensor using a kernel of degree *d* as described above.

— Otherwise, we used the following function:

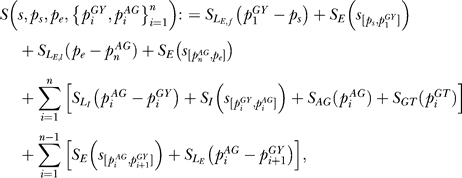
where 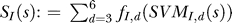 is the intron content score using the SVM intron content output *SVM_I_*_,*d*_(*s*) using a kernel of degree *d, S_AG_*(*p*): = *f_AG_*(*SVM_AG_*(*p*)) and *S_GY_*(*p*): = *f_GY_*(*SVM_GY_*(*p*)) are the scores for acceptor and donor splice sites, respectively, using the *SVM_AG_* and *SVM_GY_* output for the putative splice sites at position *p*. Moreover, 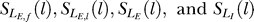 are the length scores for first exons, last exons, internal exons, and introns, respectively, of length *l*.

The above model has 15 functions as parameters. We model them as piecewise-linear functions with *P* = 30 support points at 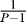 quantiles as observed in the training set. For *S_AG_, S_GY_, S_E_*_,*d*_ *,* and *S_I_*_,*d*_ (*d* = 3,...,6), we require that they are monotonically increasing, since a larger SVM output should lead to a larger score.

To determine the parameters of the model, we propose to solve the following optimization problem that uses a set of *N* training sequences *s*_1_,…*s_N_* with start points *p_S_*_,1_,…*P_S_*_,*N*_, end points *p_e_*_,1_,…*p_e_*_,*N*_, and true splicing isoforms *σ*_1_,…, *σ_N_*:

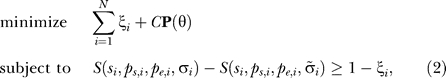
for all *I* = 1, … , *N* and all possible splicing isoforms *s~* *_i_* for sequence *s_i_,* where

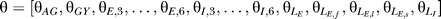
is the parameter vector parameterizing all 15 functions (the 30 function values at the support points) and **P** is a regularizer. The parameter *C* is the regularization parameter. The regularizer is defined as follows:

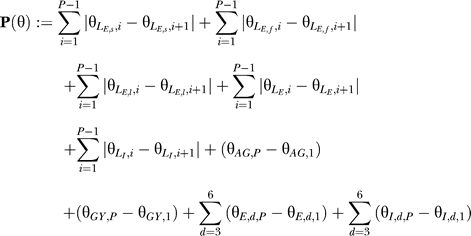
with the intuition that the piecewise linear functions should have small absolute differences (reducing to the difference from start to end for monotonic functions).

Based on the ideas presented in [19], we solve the optimization problem (2) using the cDNA sequences in the training set (these sequences were not used for training the signal and content sensors). For the model selection for parameters *C* and *P,* we use an independent validation set of cDNA sequences. The solution is found by a technique called column generation: one uses the dynamic programming based decoding algorithm (see below) to iteratively find wrong splicing isoforms with large scores. They are then added to the problem, which is then resolved. In our case, training of step 2 takes about two hours on a standard PC employing ILOG CPLEX [39] for solving the resulting linear programs.

### Decoding of splice forms.

To produce a splice form prediction 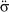 based on the splice form scoring function *S*(*s*,*p_s_*,*p_e_*,*σ*), one has to maximize *S* with respect to the splice form *σ,* i.e.,

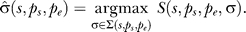
We assume that the sequence *s,* the starting position *p_s_,* and end positions *p_e_* are given. The prediction σ̂ has to satisfy certain rules, in particular that introns are terminated with the GT/GC and AG splice sites dimers and that they are not overlapping. Additionally, we require that introns are at least 30 nt and exons at least 2 nt long as well as restricting the maximal intron and exon length to 22,000 (the longest known intron in C. elegans). If one uses open reading frame information, one additionally has to make sure that the spliced sequence does not contain stop codons.

The described conditions lead to a set of valid splice forms denoted by Σ(*s*,*p_s_*,*p_e_*). Since this set grows exponentially with the length of the sequence, one cannot simply enumerate and test all possibilities. Hence, we use *dynamic programming* [40], where one defines a state model, defining valid transitions between signals that are found in the sequence. This allows us to compute the *n*-best splice forms very efficiently. (For the integration algorithm we have to generate wrong splice forms. Hence, we need to generate at least the best and *second best* scoring splice form to make sure that at least one is wrong.) In the case of the SM, the state model contains only four states: 5′ end, donor, acceptor, and 3′ end (cf. Figure 4). Every transition accepts a part of the sequence *s,* starting at position *p_s_* in state 5′ end and terminating in state 3′ end at position *p_e_*. The state's donor and acceptor require the splice site dimers at the corresponding positions. The model that takes open reading frame information into account requires 14 states to ensure that (a) no exon contains a stop codon in-frame (needs three separate intron transitions) and (b) that no concatenation of two introns can lead to a stop codon. (If the minimal exon length would be 1 nt, then a stop codon can be generated by splicing; for instance, NNT, A, and A together. It would require a more complicated model to exclude this splice form.) See Figure 5 for details.

For predicting splice forms on new sequences, one needs to compute the level 1 splice site scores and to run a decoding algorithm. Both steps together require about 40 s per 100 knt sequence on standard PC hardware (about 11 s for level 1 and about 29 s for level 2 on a 2.2-Ghz Opteron CPU). A tool for predicting the splice form for C. elegans sequences implemented in *Python* and *C++* can be downloaded at http://www.msplicer.org, licensed under GPL (General Public License, http://en.wikipedia.org/wiki/Gpl).

### Sequencing reactions.

We designed primers to amplify approximately 1,000 base pair amplicons using the program Primer 3.0 [41]. A summary of the used primers is given in the table in section 3 of Protocol S1. A typical PCR mixture consisted of 10 mM Tris-HCl, 50 mM KCl, 1.5 mM MgCl2, 200 μM dNTP, 1 unit Taq polymerase, and 1μM of each primer. Thermocycling was done in a Perkin Elmer Gene Amp 9,700 PCR machine under standard conditions consisting of an initial denaturation at 94 °C for 3 min, followed by 30 cycles of 94 °C for 1 min, 55 °C for 1 min, and 72 °C for 1 min, and a final incubation at 72 °C for 7 min. The PCR products were first confirmed on a 1% agarose gel for their expected sizes. Once the length of the products was confirmed, the products were extracted from the gel using a Qiagen Gel Extraction Kit. Sequencing reactions were set up according to manufacturers' instructions for the Big Dye Terminator chemistry (Applied Biosystems, http://www.appliedbiosystems.com). Samples were analyzed using capillary electrophoresis (Applied Biosystems, ABI Prism 3700). The software PHRED performed base calling, and vector sequences were masked with CrossMatch. Sequences containing at least 100 nonvector bases with Phred values >20 were used for further analysis. The sequences obtained were then validated by aligning them against the C. elegans genome using *blat* [22]. Once the gene identity was confirmed, we compared the gene structure of the obtained EST with our prediction and the annotation. We obtained 25 spliced mRNAs, five of which showed evidence for alternative splicing and were excluded subsequently (as in the simulation experiments).

## Supporting Information

Protocol S1 Data Preparation Protocols, Additional Results, and Primer Lists (161 KB PDF)

## Abbreviations

auPRC: area under the precision recall curve
HMM: hidden Markov model
*mSplicer*: margin splicer
nt: nucleotide
OM: sophisticated model using ORF information
POIM: positional oligomer importance matrices
RT-PCR: reverse transcription polymerase chain reaction
SM: simple model
SVM: support vector machine
UTR: untranslated region
