# Supplementary material for: Improving the Caenorhabditis elegans Genome Annotation Using Machine Learning
Source: PLoS Comput Biol. 2007 Feb 23;3(2):e20. doi: 10.1371/journal.pcbi.0030020 (PMC1808025; doi:10.1371/journal.pcbi.0030020)
Supplement: Protocol S1 — (161 KB PDF) [file pcbi.0030020.sd001.pdf]

Online Text for "Improving the *Caenorhabditis elegans* Genome Annotation using Machine Learning"

G. Rätsch, S. Sonnenburg,* J. Srinivasan,* H. Witte,
K.-R. Müller, R.J. Sommer, B. Schölkopf

* these authors contributed equally to the work.

Contents

1 Preparation of Sequence Data **2**

1.1 *Caenorhabditis elegans* 2

1.2 *C. remanei*, *C. briggsae* and *P. pacificus* 3

2 Supplementary Results **3**

2.1 List of Important Oligomers 3

2.2 Sequencing results 5

3 List of Primers **11**

1 Preparation of Sequence Data

1.1 *Caenorhabditis elegans*

1.1.1 EST Sequences

We collected all known *C. elegans* ESTs from Wormbase (1) (release WS120; 236,893 sequences) and dbEST (2) (as of February 22, 2004; 231,096 sequences). Using *blat* (3) we aligned them against the genomic DNA (release WS120). The alignment was used to confirm exons and introns. We refined the alignment by correcting typical sequencing errors, for instance by removed minor insertions and deletions. If an intron did not exhibit the consensus `GT`/`AG` or `GC`/`AG` at the 5' and 3' ends, then we tried to achieve this by shifting the boundaries up to 2 base pairs (bp). If this still did not lead to the consensus, then we split the sequence into two parts and considered each subsequence separately. In a next step we merged alignments, if they did not disagree and shared at least one complete exon or intron. This lead to a set of 124,442 unique EST-based sequences.

1.1.2 cDNA Sequences

We repeated the above procedure with all known cDNAs from Wormbase (release WS120; 4,855 sequences). These sequences only contain the coding part of the mRNA. We use their ends as annotation for start and stop codons.

1.1.3 Clustering

We clustered the sequences in order to obtain independent training, validation and test sets. In the beginning each of the above EST and cDNA sequences were in a separate cluster. We iteratively joined clusters, if any two sequences from distinct clusters match to the same genomic location (this includes many forms of alternative splicing). We obtained 21,086 clusters, while 4072 clusters contained at least one cDNA.

1.1.4 Splitting into Training, Validation and Test Sets

For the *training set* we chose 40% of the clusters containing at least one cDNA (1536) and all clusters not containing a cDNA (17215). For the *validation set* we used 20% of clusters with cDNA (775). The remaining 40% of clusters with at least one cDNA (1,560) was filtered to remove confirmed alternative splice forms.

This left 1,177 cDNA sequences for *testing* with an average of 4.8 exons per gene and 2,313bp from the 5' to the 3' end.

1.1.5 Processing the Annotation

We used the Wormbase (WS120) genome annotation. We first extracted all curated genes without annotated alternative splicing. We removed all genes that overlapped with any of the EST clusters identified above. We removed all genes with non-canonical splice sites, leaving 5,166 completely unconfirmed genes with an average of 4.8 exons per gene and 1,961bp from the start to the end of the coding region. This set was used for the comparison with our prediction method.

For the retrospective analysis we repeated steps 1.1.1-1.1.3 for ESTs and cDNAs from dbEST (as of 11/10/2005) and Wormbase (WS150). For all WS120 unconfirmed genes (see above) we identified overlapping segments of the gene with an EST or cDNA sequence match on the genome. We only considered cases where the WS150 sequences did not reveal any evidence for alternative splicing. This way identified 474 newly partially confirmed genes in 529 segments. We used 426 segments (in 379 genes) for our evaluation and the remaining sequences for model selection.

1.2 *C. remanei*, *C. briggsae* and *P. pacificus*

We repeated the steps 1.1.1-1.1.3 for the other three genomes where we started with 15,155, 2,424 and 12,428 EST sequences for *C. remanei*, *C. remanei* and *P. pacificus*, respectively. After clustering we obtained 4,395, 787 and 2,744 EST clusters. For *P. pacificus* we used a random subset of 500 clusters and for *C. remanei* and *C. briggsae* all clusters without evidence for alternative splicing or non-canonical splice sites for final out-of-sample evaluation. For retraining the second step of *mSplicer* for *P. pacificus* we used another 500 EST clusters. The splice site detectors and exon/intron content sensors have not changed.

2 Supplementary Results

2.1 List of Important Oligomers

Below is the list with the most important oligmers for discrimination of donor and acceptor splice sites (three for every length). Shown are the position relative to the splice site, the oligomer sequence and the contribution of the oligomer.

Donor Splice Sites			Acceptor Splice Sites		
pos.	oligomer	contribution	pos.	oligomer	contribution
4	G	804.82	-3	T	499.46
-1	G	405.16	-4	T	337.49
1	C	-385.33	-1	G	-273.67
4	GT	894.76	-4	TT	658.72
3	AG	721.65	-3	TT	472.53
1	TA	608.19	-1	GA	-324.98
3	AGT	668.43	-1	GAG	-324.98
0	GTA	635.21	-5	TTT	305.62
-1	GGT	609.74	-5	ATT	297.98
-1	GGTA	525.98	-4	TTGC	245.98
1	TGAG	459.47	-2	AGAG	-244.09
-2	AGGT	447.12	-4	TTCC	219.95
0	GTGAG	472.91	-4	TTGCA	251.87
1	TGAGT	407.76	-4	TTCCA	230.07
0	GTAAG	396.98	-4	TTACA	223.25
0	GTGAGT	417.61	-4	TTGCAG	251.87
39	TTTCAG	339.38	-4	TTCCAG	230.07
43	TTTCAG	321.05	-4	TTACAG	223.25
-1	GGTTTGT	226.82	-5	TTTCCAG	161.39
-1	CGTAAGT	189.79	-4	TTTCAGC	147.98
0	GTGAGTT	187.91	-4	TTACAGA	142.28
-10	TTAGGCTT	150.62	4	TTAGGCTT	108.79
-2	AGGTAAGT	-145.69	5	TAGGCTTA	106.05
-2	AGGTAGGT	-139.69	-5	TTTCCAGC	100.59
-10	TTAGGCTTA	124.23	4	TTAGGCTTA	123.75
-11	CTTAGGCTT	111.37	5	TAGGCTTAG	94.86
-6	TTTCAGGTA	-97.74	3	CTTAGGCTT	94.21
-10	TTAGGCTTAG	88.86	4	TTAGGCTTAG	111.70
-11	CTTAGGCTTA	82.61	3	CTTAGGCTTA	105.58
-8	AGGCTTAGGC	79.02	5	TAGGCTTAGG	79.96
-13	GGCTTAGGCTT	91.50	4	TTAGGCTTAGG	96.24
-10	TTAGGCTTAGG	84.07	3	CTTAGGCTTAG	94.68
-14	AGGCTTAGGCT	71.61	5	TAGGCTTAGGC	56.37

2.2 Sequencing results

Out of the set of completely unconfirmed genes in WS120, we randomly selected a set of 24 genes where our predictions significantly differed from the annotation (accession ids: B0432.7, C05A9.3, C18F10.9, C18G1.8, C35E7.7, F02D10.3, F07E5.6, F21C10.9, F26D2.12, F40H7.5, F49C5.1, F49H6.10, F59H6.1, H17B01.3, K04E7.1, K08D10.9, M03E7.3, M4.1, R02C2.4, T04C10.3, T12C9.7, T14G12.6, Y32B12C.2, and Y46H3A.4). To do this we computed the first ten best predictions of *mSplicer* (the ten paths with highest scores) and only included it in our set if the annotation did not match any of our ten predictions. This way we look a particularly biased hard set of genes. Later we performed the sequencing experiments showing that in 15 out of 20 cases we predicted exactly correct. The remaining four cases showed evidence for alternative splicing. In the table below we display the splice forms annotated in WS120, our prediction and the ones derived from the sequencing results.

Predictions, WS120 Annotation and sequencing results

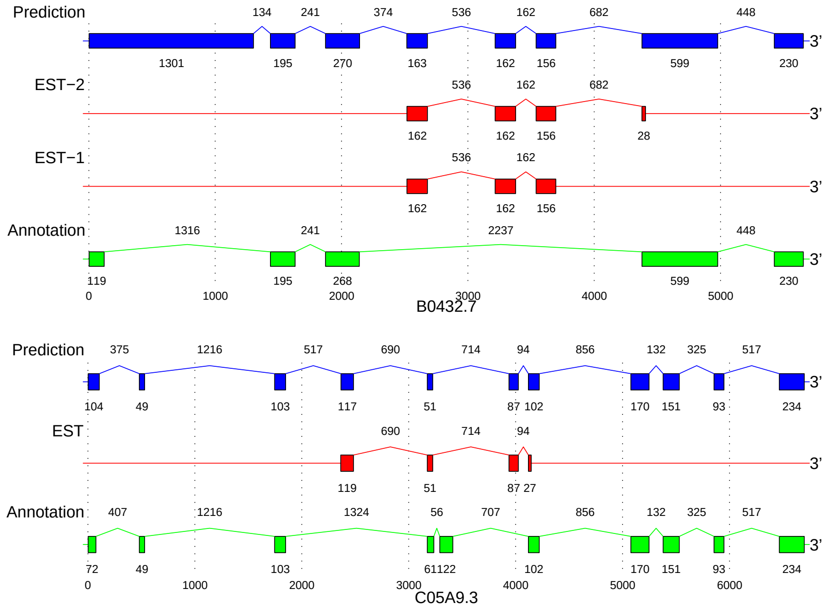

cont'd on next page

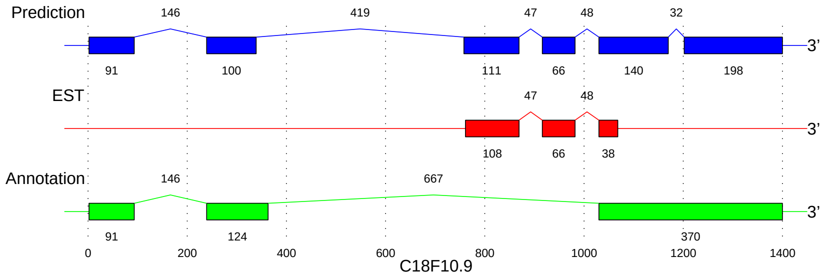

C18F10.9

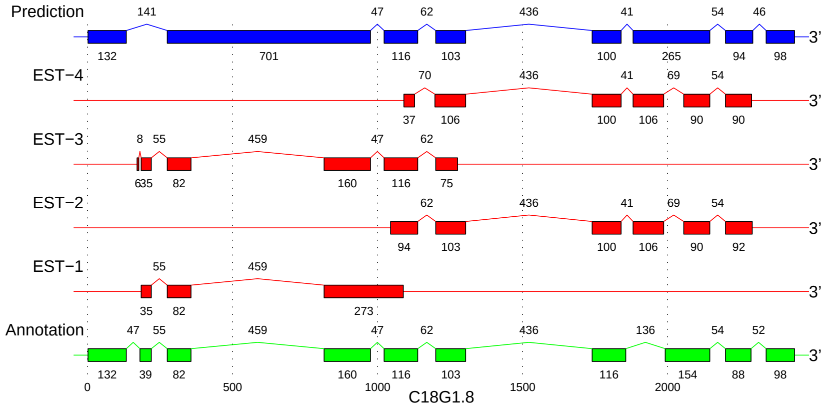

C18G1.8

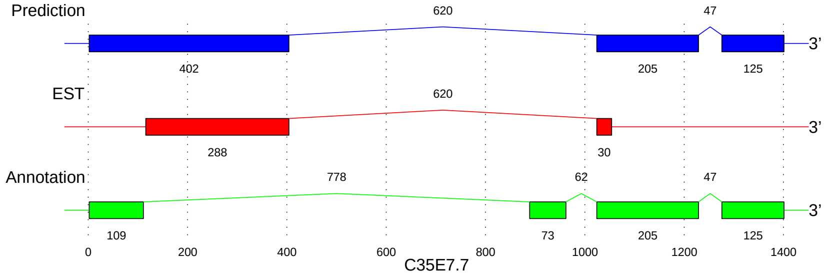

C35E7.7

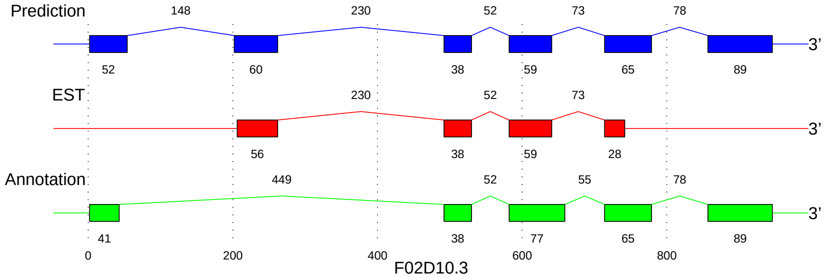

F02D10.3

cont'd on next page

Predictions, WS120 Annotation and sequencing results

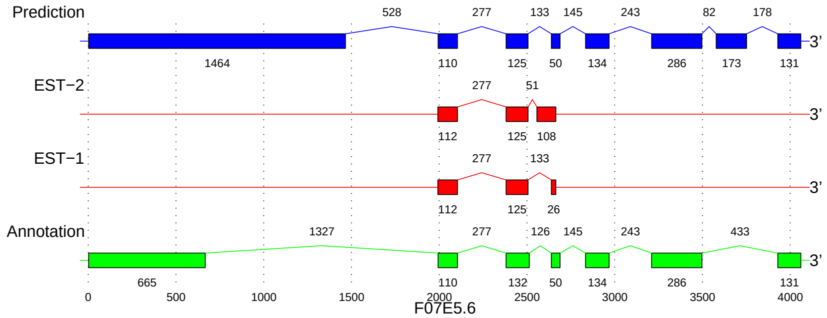

F07E5.6

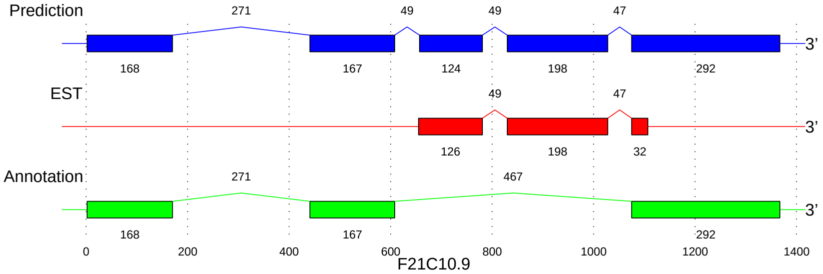

F21C10.9

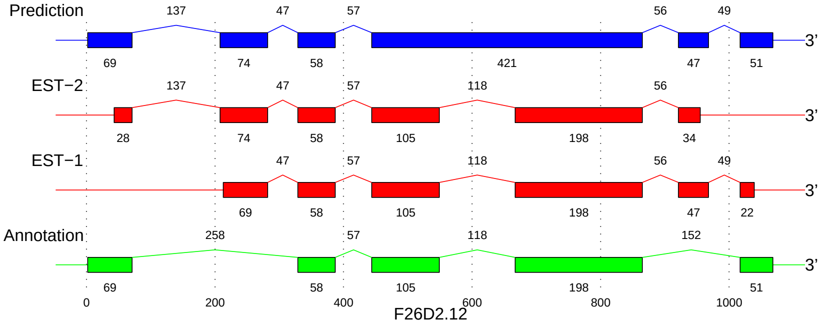

F26D2.12

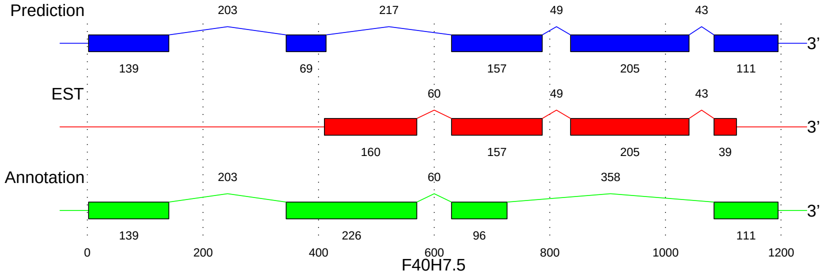

F40H7.5

cont'd on next page

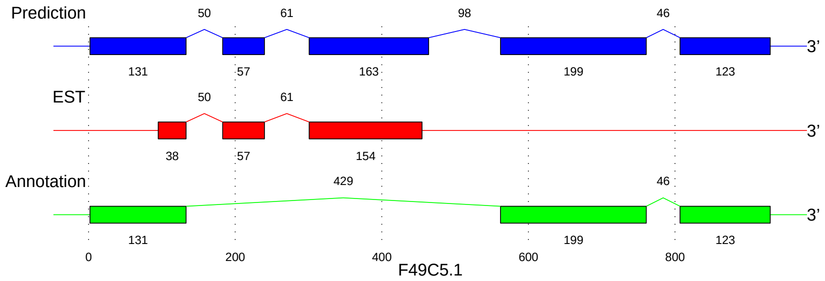

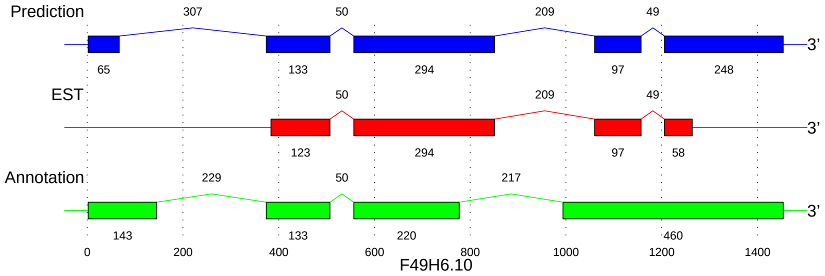

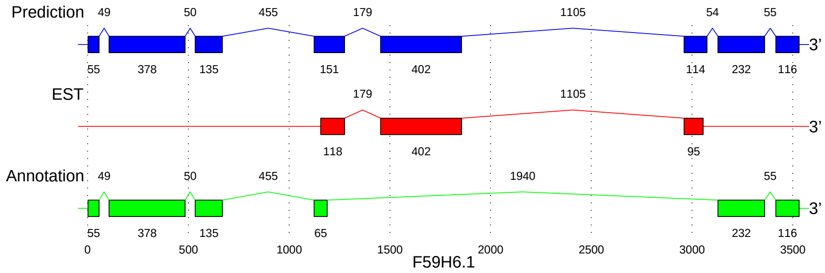

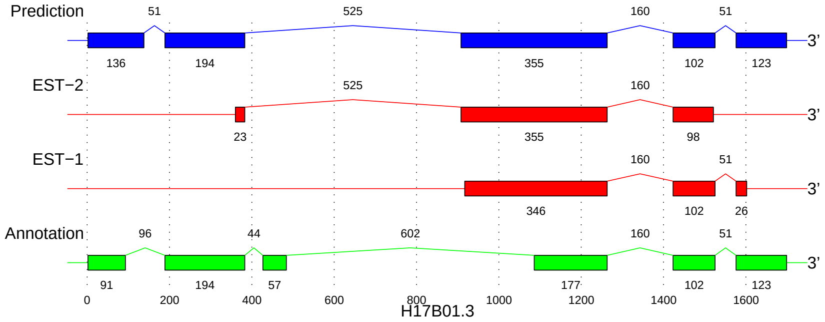

cont'd on next page

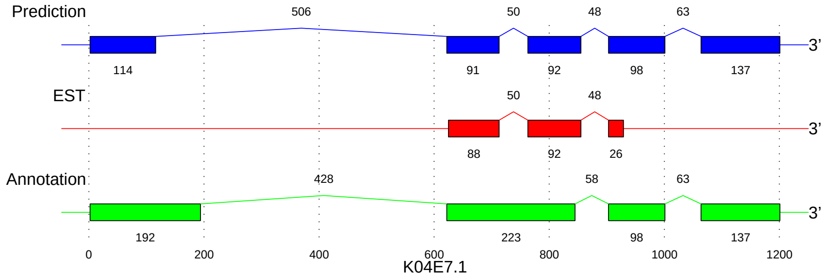

K04E7.1

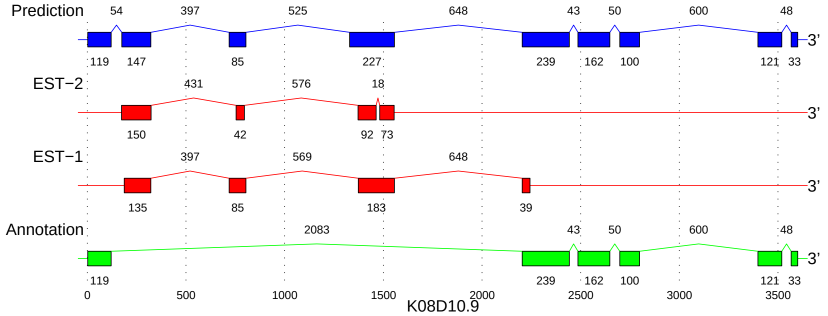

K08D10.9

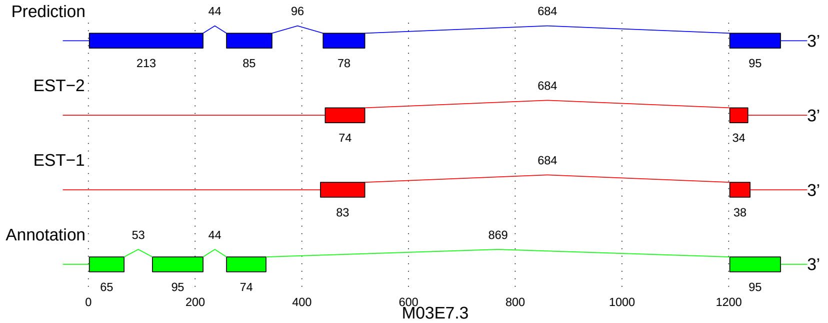

M03E7.3

cont'd on next page

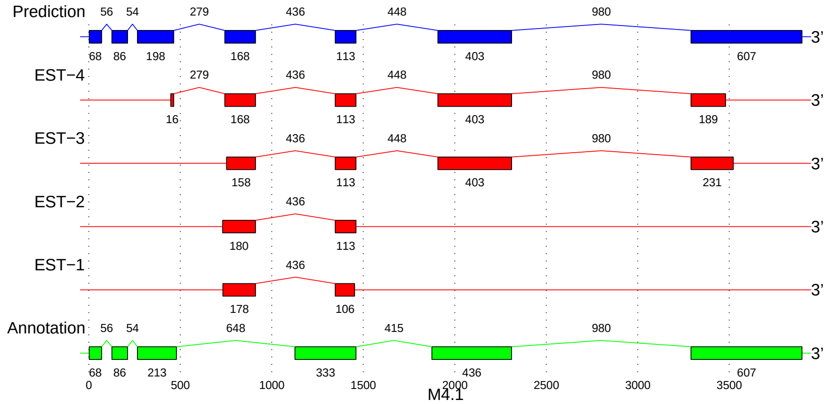

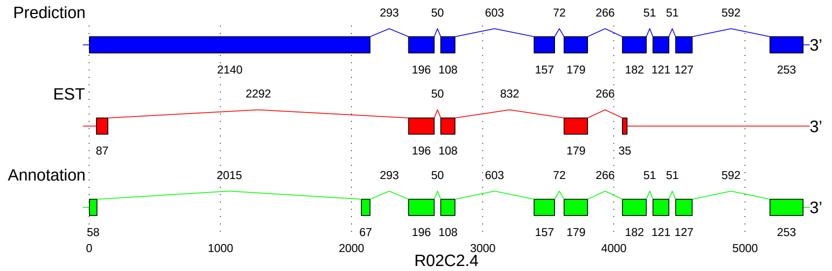

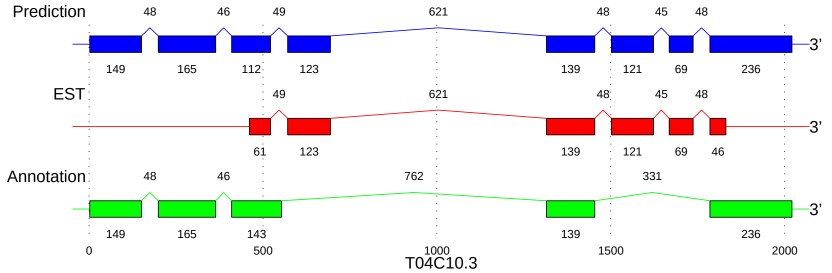

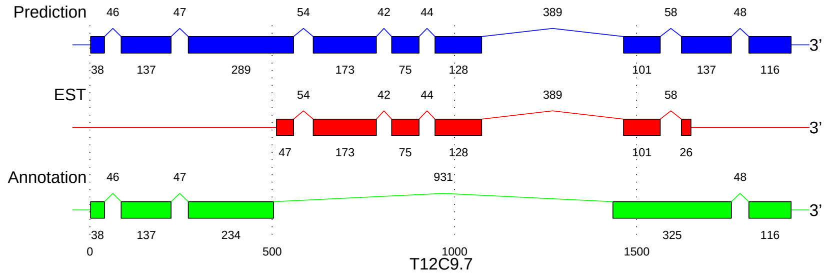

cont'd on next page

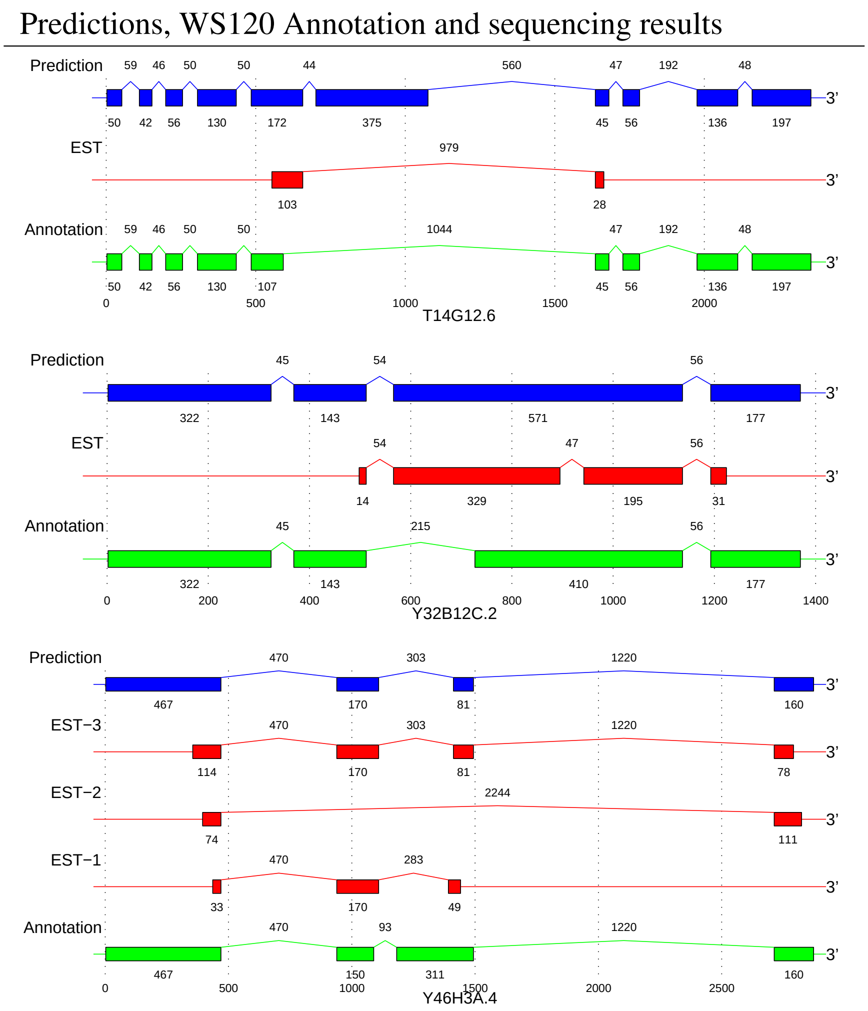

Table 1: The illustrations show the annotated (green), predicted (blue) and newly confirmed (red) splice forms of 25 *C. elegans* genes that were unconfirmed in WS120.

3 List of Primers

For every sequencing experiment we designed two sets of nested primers. The outer primer pair was used for PCR amplification and the inner primer pair for sequencing from both ends. In a few cases we have designed a few more primers.

Only in 31 out of 44 experiments we obtained sequencable PCR products. 7 cases were excluded since no splicing was observed (to exclude contamination with DNA). Hence, we could only consider 24 sequences (marked with + or ∗) for further analysis. This suggests that in the unconfirmed part of the annotation many genes include intergenic regions resulting in primers pairs matching two separated mRNA sequences and therefore no PCR product. We found four genes (marked with +) which show evidence for alternative splicing (excluded from our analysis).

Primer Nr.	Primer Sequence	Gene name
GR5	TTTTATCGCAGATTGTCATCG	R02C2.4*
GR6	GGATTTGGTTTTCTGGATGCT	R02C2.4
GR7	CAGATTGTCATCGAACTTTATCG	R02C2.4
GR8	TATCGTCTCCGGGCTCAG	R02C2.4
GR33	CATCACTCATTCCAGCCCC	T12C9.7
GR34	CGTTTCGCGGAGAACTGT	T12C9.7
GR35	CATTCCAGCCCCTCATACTCT	T12C9.7
GR36	TGTCGACGGAGTTTGATCTAC	T12C9.7
GR45	TGTTGTCAGTTCTTGCTTTCC	F26D2.12*
GR46	TCCGCATACATACCCAGTG	F26D2.12
GR47	TTCTTGCTTTCCTACTCAGCAA	F26D2.12
GR48	CAGTGGGATCAGCTCGGA	F26D2.12
GR65	GAGCACAGTAAACTTGGTGGC	F40G9.5
GR66	GATTGAACGGGAGCCATGT	F40G9.5
GR67	GTAGGCTCCGTTGCTATCGTT	F40G9.5
GR68	AGCCATGTGGGAAATTGGAT	F40G9.5
GR69	GCTTTCTCGCCATGTATTGTC	M03E7.3
GR70	ATCTACCGGTGGCATTTCC	M03E7.3
GR71	ATTGTCTATGGTGGTTCGGTG	M03E7.3
GR72	TTCCAATTGGGATTTGTCATC	M03E7.3
GR453	CATTTCGTTGGCGATGCTACTC	M03E7.3
GR455	CTCTTTACATTGAAAATGAACA	M03E7.3
GR89	TTCCACCAAACAGTCCAGAAC	T14G12.6*
GR90	TGTTACGGTCGATGTCTCCAT	T14G12.6
GR91	GAACAAATTGTCCTTGGGTTG	T14G12.6
GR92	CATTGCAGGTGTTGTCATCAT	T14G12.6
GR437	CCAATGTAGTCATGACAACTG	T14G12.6

cont'd on next page

Primer Nr.	Primer Sequence	Gene name
GR438	GACCACTGACGCCAAATCTGG	T14G12.6
GR439	CACGTGGCTGCACTAATTTTGC	T14G12.6
GR440	GACTCGGACGGTTGCATTGAGC	T14G12.6
GR97	CTTTCCATTTTTGCACATGAC	F49C5.1
GR98	TGACGATATTCCAGTTGAGCA	F49C5.1
GR99	TTTTGCACATGACAAAGTATCGT	F49C5.1
GR100	TGAGCACTCGAAACTGTTGGA	F49C5.1
GR109	TATGGAGATTCACCCGACTCA	C37E2.3
GR110	GAAATCAAAGCATAACGCAGC	C37E2.3
GR111	CAAAGGAGTTGTATATTTTCCGA	C37E2.3
GR112	GCAGCTAGCCAAACGACAC	C37E2.3
GR121	TGAAGGGAGAGGAAGCAATTT	F07C3.2
GR122	CCTGATTGGCAATTCTCCATA	F07C3.2
GR123	TTTCAATTGTGTTCAGTTTTTCA	F07C3.2
GR124	GGTACAGTTGGTTTCGGCATA	F07C3.2
GR125	TGCCATGTACATTCAGCACC	F41H8.3
GR126	GAGAGCGTTCCAAAATGATTG	F41H8.3
GR127	ACATTCAGCACCGATATGAGC	F41H8.3
GR128	TGGAAATACTGATAAGGAGCACA	F41H8.3
GR133	CTTTCATGAACACCCTTGTCA	F40H7.5*
GR134	TTGTTTCCCTCATTTTGACAGT	F40H7.5
GR135	ACCCTTGTCAATGAAATGCTG	F40H7.5
GR136	TTTGTTTTCACACTCCTGATTGA	F40H7.5
GR137	CAATGGACTAGCCGATTTCC	K08D10.9+
GR138	GAATCACAACAACAGAACCGC	K08D10.9
GR139	TCCGGAATGATGATGAATTTG	K08D10.9
GR140	CAGAACCGCAAAGAGAGAATG	K08D10.9
GR165	TTTTGGAGGTGGAAATCATGT	T13B5.7
GR166	GTTGTATTGCCCCATGTTGTT	T13B5.7
GR167	TGGAAATCATGTTGGAGGAGT	T13B5.7
GR168	TGTTGTGTAGACGGTTTCATCA	T13B5.7
GR177	TACATTGATGATTGGCGTCAC	T07C5.4
GR178	AAGCGATTAAATCACGACCG	T07C5.4
GR179	TCACGACGAACATTGTTTCAA	T07C5.4
GR180	ACCGGTGTTTGATAAACCAGA	T07C5.4
GR193	GGCGTGGAAATTGTGGAA	M4.1*

cont'd on next page

Primer Nr.	Primer Sequence	Gene name
GR194	TGTTGGAGGATAGGATTGACA	M4.1
GR195	AAATTGTGGAAAACGCGAAT	M4.1
GR196	TGACAATTGTGCTTCCAGTGA	M4.1
GR446	GACTCTTCCGACGATTCAGATG	M4.1
GR448	GATTCAGATGACTGAGCAAATC	M4.1
GR209	GGACACCACTAGTTCTTCGACC	Y53G8AL.3
GR210	GTCTTCCTATTTGCTCCGCAC	Y53G8AL.3
GR211	CTTCGACCACTGAAGTTCCTG	Y53G8AL.3
GR212	ACTGCTCGGATTTGGAGGTT	Y53G8AL.3
GR217	AAGGCAGTGAACCTCACAAAG	Y69H2.7
GR218	GCCATTTGGAAGAGCAGGT	Y69H2.7
GR219	CCGTCACTCAAAGCATCAATA	Y69H2.7
GR220	CAGGTGCTGGTTCATTTGG	Y69H2.7
GR225	CGTTAGTTTTATTGAACGAATGC	C35E7.7*
GR226	TCTGGATATTCGGTTTGAAGC	C35E7.7
GR227	ATGCGCACTTTCCAGTTCTTA	C35E7.7
GR228	CAAATGTTGGTTGTCTGATGC	C35E7.7
GR442	GCTTATCATCATAGGTTTCTGC	C35E7.7
GR444	CTGCTTGTCCGTCATAATACC	C35E7.7
GR229	GGCTCAAGCAATGTCTCGTAT	F21C10.9*
GR230	TGATGAATTTGCGTAAAGGTG	F21C10.9
GR231	GGAAAGACTTGGTTCTTGGCT	F21C10.9
GR232	CGTAAAGGTGGCAAATTTTGAA	F21C10.9
GR233	CATTGGAACATTGGGCAAAC	B0432.7*
GR234	GAGTTGTTGAAGGGAGCAGAA	B0432.7
GR235	TTGGGCAAACGAGCTTATATC	B0432.7
GR236	GAGCAGAAAGCCAGGAGAAG	B0432.7
GR253	CAAAGCCAGGATTCACTGAGA	F07E5.6$^+$
GR254	GAAACTCCTCCTTGAGCCAAA	F07E5.6
GR255	TTCACTGAGAAACTTTGGATCG	F07E5.6
GR256	CGACTTGTTGAACTTGTGTTGG	F07E5.6
GR257	CACTTCCGGATTTGCAATG	K04E7.1
GR258	CGCTTCGATAGGGGGTAATA	K04E7.1
GR259	GTCCTCCAGCACTCCATTG	K04E7.1
GR260	TGCAAATGCATTCTCAATACAA	K04E7.1
GR265	CCTCATTTCAATAGCTGTCGC	Y32B12C.2*

cont'd on next page

Primer Nr.	Primer Sequence	Gene name
GR266	TGAATAGTTCCGTTGGCAAGT	Y32B12C.2
GR267	GTCGCCATGGCAGTTCTAC	Y32B12C.2
GR268	CAAGTGGTACAAACGCATGAA	Y32B12C.2
GR457	GTATTATCGAAAGTATCAGAAG	Y32B12C.2
GR458	TCCTTCATCATTTTTATATGT	Y32B12C.2
GR459	AGTATCAGAAGTTCAAATTTGG	Y32B12C.2
GR460	TGTAAATTTGATAAGGTATAG	Y32B12C.2
GR277	TCCAGGAAGTTCAAATCATCAA	C18F10.9*
GR278	TGTCTTCTGATTGGTGGTTGC	C18F10.9
GR279	TCAAATCATCAAGGATGAACCA	C18F10.9
GR280	TTGCCATTGGGAATTTGAGT	C18F10.9
GR281	CGGAAGCTCACACAAGAATCC	Y22D7AL.12
GR282	AAAACGGCGGTTGTTTCG	Y22D7AL.12
GR283	CACAAGAATCCGCTACTCG	Y22D7AL.12
GR284	TTCGGAAGACCAGTTAGGG	Y22D7AL.12
GR313	TCGTCGGAATCCTTCACCT	F59H6.1
GR314	CTCAAGCTTGTGAGCCAGG	F59H6.1
GR315	CCGATTATAAAATGCCACTTCC	F59H6.1
GR316	GAGCCAGGTAGAGAATTGCGT	F59H6.1
GR317	TCCCGAAAGATCAGAATAGAGG	Y46H3A.4+
GR318	GGTGCACACCGTATTTCCATA	Y46H3A.4
GR319	CAGAATAGAGGATCGTTTCATCA	Y46H3A.4
GR320	CCATATGGATCGTAGTAGGCAGA	Y46H3A.4
GR461	GACCTGGCTGAGGCACACGATG	Y46H3A.4
GR462	CAGCAACAGCAACCACCTTCC	Y46H3A.4
GR463	GAGCTTGTTCCGGATTCGTG	Y46H3A.4
GR464	CCTTCCGAGCAGGAGCACAAC	Y46H3A.4
GR321	CGGAATTCTCAGAAGCCCATA	C18G1.8+
GR322	GTGTCCAGTGAGGCAAGAAAT	C18G1.8
GR323	AAGCCCATATCCTTGGCTTAT	C18G1.8
GR324	TCATAAGGCAGTAATTGTCCG	C18G1.8
GR333	CTTGACTTTTCATATATTCCCGA	F49H6.10
GR334	AAGGCGTTGTGATAACATCAGT	F49H6.10
GR335	AACGAATTCATCTGTGGCATC	F49H6.10
GR336	AATGGCCATCCAAATGTGATA	F49H6.10
GR349	CAAATCAAATTTTCAGCGCAC	F47C12.8

cont'd on next page

Primer Nr.	Primer Sequence	Gene name
GR350	ACCAGGAGTTTTCGTCTCGTT	F47C12.8
GR351	GCACCCAAGAGGGGACAT	F47C12.8
GR352	ATGAAGGGAGCTTTTTGTCGT	F47C12.8
GR365	GTTACAGCACGCGTCATTTTT	C06A1.2
GR366	GAGCTCAGTGCATTCTGTCG	C06A1.2
GR367	CGTCATTTTTAGGGCTTGATG	C06A1.2
GR368	GGCCATCCACATAGTGTCATT	C06A1.2
GR373	GAGGCCAGCAAAATCAACA	F02D10.3*
GR374	ATCGTCCACTGCGATATTCAT	F02D10.3
GR375	CAAAATCAACAGCGAGGACA	F02D10.3
GR376	TGTCCTGGTACTCATAATCGAAA	F02D10.3
GR397	GCCGCTATCGGATAATGATG	T03E6.2
GR398	GAAGACTATCAGACTGCCCACC	T03E6.2
GR399	TGTGATTATGCTTTACTCGCTGA	T03E6.2
GR400	CCACCGGGAAGTACATTGTTA	T03E6.2
GR405	CGCGCATATGTCTTTTTCC	F47F2.2
GR406	GCGCGCGTCATTATTTCT	F47F2.2
GR407	TGTCTTTTTCCAGTGGTAGTGG	F47F2.2
GR408	ATTATTTCTCACGGCTTCGTC	F47F2.2
GR413	TTCGTTCAGCCTATGAACTTTG	F49A5.7
GR414	CCTCCTTCTCTCATACAATCGAA	F49A5.7
GR415	CTTTGTTTACGAGCTTCCGGT	F49A5.7
GR416	CAATCGAAATCAGCATTGTCT	F49A5.7
GR417	GACAAAGGTTACAGCGACAGC	C05A9.3*
GR418	TGTCTACGTTGAGCAAGATCC	C05A9.3
GR419	CAGCGACAGCAAAGTGGTC	C05A9.3
GR420	GCAAGATCCGTCAATGTGTTT	C05A9.3
GR449	GGATATTGTATTGAACGTTGG	F56H1.2
GR450	GGTGGTATGCCAACTCGAACG	F56H1.2
GR451	ACGTTGGACGTGGACATGCG	F56H1.2
GR452	TATGCCAACTCGAACGCGATGC	F56H1.2

References

[1] Harris T, Chen N, Cunningham F, et al. (2004) Wormbase: a multi-species resource for nematode biology and genomics. Nucl Acids Res 32. D411-7.

[2] Boguski M, Tolstoshev TLC (1993) dbEST–database for ”expressed sequence tags”. Nat Genet 4:332–3.

[3] Kent W (2002) Blat–the blast-like alignment tool. Genome Res 12:656–64.
